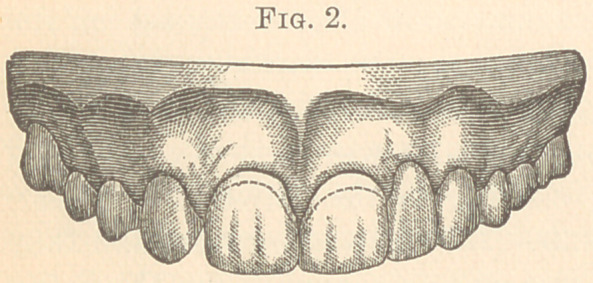# Two Interesting Cases

**Published:** 1889-11

**Authors:** L. C. Taylor

**Affiliations:** Hartford, Conn.


### TWO INTERESTING CASES.[1]

BY L. C. TAYLOR, D.D.S., HARTFORD, CONN.

Two years ago I presented models to many members of this society (Massachusetts), asking their advice as to the best method of treatment. The history of the case is as follows: The patient, the daughter of a well-known clergyman, was very desirous of securing the best cosmetic effects, and did not wish the pulps destroyed, if it were possible to save them alive. She said that she had consulted several other dentists, who had advised "letting alone or cutting off and crowning." The deformity, caused by a serious illness occurring previous to the second year, consisted of abnormally short superior central incisors. These teeth, when erupted, were imperfectly formed, and soon after decay set in, the pits of which were mostly located near the cutting edge. The cutting edges gave way, and the teeth were shortened fully one-third their normal length. The deformity was further aggravated by an unusually long upper lip, which gave the patient the appearance of having lost her front teeth. In ordinary conversation they did not show at all. When the case was presented to me, the decay had ceased and the pulps were alive and healthy. A cast, taken at that time, shows more plainly than can be described the condition of the mouth when I first saw the patient. (See Fig. 1.) The laterals had not made their appearance, but the space had nearly closed on the right side. I at first thought of drawing the centrals down, but gave the idea up as impracticable, and finally decided to put shell-caps on the two centrals, and fill the space left by the non-eruption of the left lateral by inserting an artificial clasp plate. This tooth might have been attached on a bridge to the central, but I was afraid of loosening it, as we did not know positively how well the roots were developed, the faulty development of the crown leading to the inference that the root might have been similarly affected. The operation consisted in bevelling off the natural

FIG. 1.

[1] Read at the semi-annual meeting of the Massachusetts Society, Boston June 6, 1889.

tooth as much as was safe without approaching too near the pulp. We then took an impression, and made a metal die, to which gold caps were fitted, extending well up under the gum. We then selected some of Ash's plate teeth with long pins, grinding out that portion above the pins until quite thin, and apparently well fitted to the face, then bevelled off the tooth, allowing the pins to come to the gold cap over the natural tooth. We then ground the end and face of the artificial tooth until apparently about right, soldered it to cap, and finished with pure gold band around sides to make good joints. We next cemented the crowns to the natural teeth with oxyphosphate; finding them a little long, we still further ground from face and ends, after which we polished the face of tooth. We are indebted to Ash & Sons for teeth that enable us to do this kind of work, as they can be finished in the mouth. The work, as completed, is shown in Fig. 2. The dotted lines show how far up the porcelain face extended. I believe the expression is about perfect. The object we had in view was to save the pulps of the teeth. These teeth are now alive and as healthy as any in the audience. I do not believe we are justified in destroying the pulps of teeth where it is possible to save them alive.

Fig. 2.

The second case was that of a young patient, eight years of age, with the tip of a central incisor broken off. It is an instance where ossification is somewhat in excess of ordinary cases. This (presenting the broken portion of natural tooth) shows you just how much was gone. The pulp was not exposed, owing to excessive ossification. This tooth was treated in the same way as the previous case; and while the operation may be criticised, it seemed the better thing to do, as we are all well aware that, as the root is not fully formed at the age of eight, it must prove almost anything but satisfactory to destroy the pulp.

## Figures and Tables

**Fig. 1. f1:**
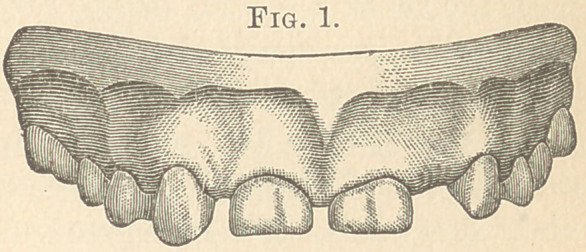


**Fig. 2. f2:**